# Intelligent Vehicle Decision-Making and Trajectory Planning Method Based on Deep Reinforcement Learning in the Frenet Space

**DOI:** 10.3390/s23249819

**Published:** 2023-12-14

**Authors:** Jiawei Wang, Liang Chu, Yao Zhang, Yabin Mao, Chong Guo

**Affiliations:** 1 College of Automotive Engineering, Jilin University, Changchun 130022, China; wjw20@mails.jlu.edu.cn (J.W.); chuliang@jlu.edu.cn (L.C.); zyao18@mails.jlu.edu.cn (Y.Z.); maoyb21@mails.jlu.edu.cn (Y.M.); 2 Changsha Automobile Innovation Research Institute, Changsha 410005, China

**Keywords:** autonomous driving, deep reinforcement learning, behavior decision making, trajectory planning

## Abstract

The complexity inherent in navigating intricate traffic environments poses substantial hurdles for intelligent driving technology. The continual progress in mapping and sensor technologies has equipped vehicles with the capability to intricately perceive their exact position and the intricate interplay among surrounding traffic elements. Building upon this foundation, this paper introduces a deep reinforcement learning method to solve the decision-making and trajectory planning problem of intelligent vehicles. The method employs a deep learning framework for feature extraction, utilizing a grid map generated from a blend of static environmental markers such as road centerlines and lane demarcations, in addition to dynamic environmental cues including vehicle positions across varied lanes, all harmonized within the Frenet coordinate system. The grid map serves as the input for the state space, and the input for the action space comprises a vector encompassing lane change timing, velocity, and vertical displacement at the lane change endpoint. To optimize the action strategy, a reinforcement learning approach is employed. The feasibility, stability, and efficiency of the proposed method are substantiated via experiments conducted in the CARLA simulator across diverse driving scenarios, and the proposed method can increase the average success rate of lane change by 6.8% and 13.1% compared with the traditional planning control algorithm and the simple reinforcement learning method.

## 1. Introduction

The realm of intelligent driving necessitates the establishment of safe and efficient interactions between vehicles and the various obstacles encountered within the road environment. To fulfill the driving tasks prescribed by the operator, an autonomous driving system typically comprises four fundamental modules: perception [1], decision making [2], planning [3], and control [4]. It is worth noting that sound behavior decision making and trajectory planning are pivotal components that chart a secure and rational course for the vehicle, ultimately underpinning the realization of intelligent driving [5]. Behavior decision-making and trajectory planning processes are significantly influenced by various critical factors. Chief among these are the static attributes of the road and lane infrastructure, alongside the dynamic attributes of other vehicles acting as obstacles [6]. In this context, the static attributes, including road layouts and lane configurations, can be derived via the integration of high-definition maps (HD Map) in conjunction with precise vehicle positioning [7]. Concurrently, dynamic obstacle information primarily originates from onboard sensors, which provide real-time data regarding the behavior of surrounding vehicles [8].

Presently, numerous research initiatives are focused on tackling the complex intricacies related to the decision-making processes and planning methodologies employed by intelligent vehicles. Significant efforts have been directed towards forecasting essential variables such as the speed of surrounding vehicles. For instance, in Ref. [9], predictions are formulated utilizing the hidden Markov model, while Ref. [10] employs the time-space interval topology method. These predictions are subsequently mixed to steer the planning and control of vehicle motion. The evolution of AI technology, coupled with enhancements in hardware computing resources, has propelled learning-based approaches to the forefront of research interests. In this context, Refs. [11,12,13] embraces deep learning methodologies. Reference [11] leverages an attention-based convolutional neural network (CNN) model to discern traffic flow characteristics from a bird’s-eye perspective of the road environment. These extracted features inform decisions regarding the next course of action for the intelligent vehicle, including predictions of lane change timings when necessary. Reference [12] introduces a lane change decision model grounded in deep belief networks (DBN) and a lane change implementation model based on long short-term memory neural networks (LSTM). Together, these models holistically characterize and validate the decision and execution of vehicle transitions. In Ref. [13], a more comprehensive model called Transformer is adopted to model both the intent decision and trajectory prediction. This integrated approach outperforms the performance of CNN and LSTM in terms of intent prediction. Reinforcement learning methods bifurcate into two categories: those operating within a discrete action space and those functioning within a continuous action space [14,15,16]. In the discrete action space category, Ref. [17] incorporates the deep Q network (DQN) to determine the behavior space, considering whether to initiate lane changes, and the state space, reflecting personalized driver style parameters. This approach thereby accommodates the influence of driver preferences on intelligent vehicle behavior decisions. Building upon DQN, variants such as Ref. [18] employ the double deep Q network (DDQN) to mitigate DQN error overestimation, while Ref. [19] introduces duel double DQN (D3QN) to introduce the value of lane change benefits, optimizing lane change decision selection for enhanced training stability. In the continuous action space category, Ref. [20] leverages the proximal strategy optimization (PPO) and hybrid reward mechanism to hierarchical plan vehicle behavior and motion. The strategy’s advancements are validated using the traffic flow simulation software SUMO (version 1.15). Reference [21] adopts the soft actor–critic (SAC) mechanism, with the state space structured around vehicle and environmental information. The action space encompasses temporal and velocity parameters, integrating trajectory planning into the reward function to enhance planning efficiency. References [22,23] employ deep deterministic policy gradients to train strategies within a continuous action space. In this approach, the policy network directly outputs actions, thereby determining the timing of intelligent vehicle lane change decision. Furthermore, variant algorithms, such as the Twin Delayed Deep Deterministic Policy Gradient (TD3) algorithm [24], Distributed Distributional DDPG (D4PG) [25], and Asynchronous Advantage Actor–Critic (A3C) [26], have been successfully applied to tasks associated with behavior decision making and planning. These algorithmic variations have demonstrated remarkable efficacy in these domains.

In the overarching framework of intelligent driving [27], the task of trajectory planning resides downstream of behavior decision making and shoulders the responsibility of translating extended decision-making objectives into specific vehicle driving paths within predefined temporal windows [28]. These driving trajectories encapsulate the vehicle position and velocity data at discrete time intervals [29]. Moreover, it is imperative that they adhere to the constraints imposed by kinematics and vehicle dynamics [30]. When the behavior decision-making task provides the state information at the trajectory’s terminal point, combined with the state information at the initiation of planning, the trajectory specifics can be elucidated via optimization. An exemplary traditional vehicle trajectory planning technique, grounded in the natural coordinate system and often referred to as the Frenet coordinate system [31], has been successfully employed in autonomous driving initiatives, including Apollo [32,33,34], yielding commendable outcomes. Differing from the conventional Cartesian coordinate system, the Frenet coordinate system dictates vehicle coordinates based on the distance ’s’ traveled along the road’s centerline and the lateral offset ’l’ perpendicular to the road’s centerline. Consequently, the road centerline, as provided in HD maps [35], serves as the foundational path. The vehicle’s driving trajectory is then expressed within the Frenet space. This framework facilitates an intuitive representation of the relationship between the road and vehicle’s location, thereby enhancing model interpretability.

In the realm of intelligent driving, there is an abundance of rich datasets originating from real vehicle sensors and trajectories, which find extensive application in perception and the decision-making processes of intelligent vehicles. Real datasets offer the advantage of being derived from actual vehicle testing, thereby capturing the authentic characteristics of real-world driving scenarios. However, it is worth noting that most of these datasets obtained from real vehicle operations predominantly encompass sensor information, trajectory records, and obstacle movements, while often lacking semantic-level definition of traffic scenarios. Real datasets are typically collected during routine driving on standardized roads, with limited representation of accident scenarios or sudden road conditions. Extracting such rare records from real-world driving necessitates significant manual effort. Acknowledging this limitation as inherent to real datasets, this paper advocates for the generation of scenario data within the driving simulator. Simulators possess the capability to model a comprehensive spectrum of driving scenarios, encompassing both typical and unexpected situations. Furthermore, simulators provide the flexibility to obtain various scene attributes, such as dynamic obstacle trajectories and lane features. As a result, diverse scenario states can be generated and acquired more readily compared to real datasets. To address this need, this paper selects the CARLA simulator (version 0.9.11)  [36] for scenario data generation. CARLA offers the advantage of permitting the specification of environmental vehicle information. The autonomous driving module integrated with CARLA can achieve a certain level of autonomous driving based on predefined rules, although its driving proficiency falls short of human expertise. Nevertheless, it supplies invaluable Ground Truth data, which is indispensable for dataset compilation. CARLA also provides HD map files in the Opendrive [37] format for simulated scenarios, enabling easy access to road network connectivity and scene-specific information, including road curvature, lane configuration, and path details.

**Figure 1:**
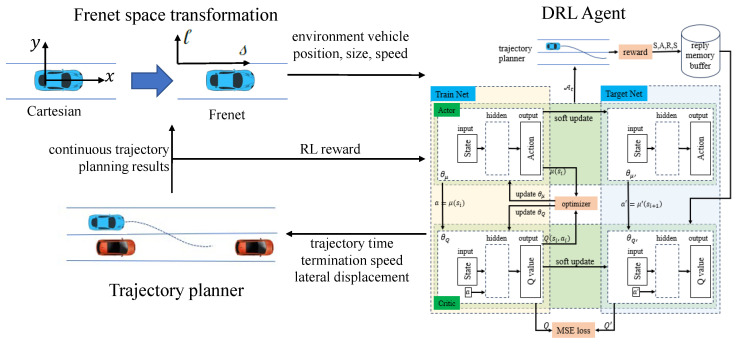
Framework of proposed method.

As mentioned in the above literature, a variety of methods have been applied for vehicle behavior decision-making and trajectory planning tasks. A common idea is to make decisions on vehicle behavior (acceleration, deceleration, or lane change) according to the learning-based method, while vehicle trajectory is planned and controlled according to the decision-making results, which decouples behavior decision from trajectory planning. Although the modularization is realized to a certain extent and the overall interpretability of the model is improved, there are also situations where behavior decision is prone to inefficiency or unsafe trajectory decision making. Therefore, it is necessary to improve the efficiency and security of decision making and planning without losing the interpretability of the model. Based on this need, this paper introduces an approach for intelligent vehicle behavior decision making and trajectory planning, which leverages the Deep Deterministic Policy Gradient (DDPG) technique within the Frenet space framework. The proposed method is structured into two hierarchical layers. The upper layer employs Deep Reinforcement Learning (DRL) via the DDPG algorithm to make behavior decisions for the intelligent vehicle. The DDPG model takes into consideration various input parameters, such as the relative spatial positioning, dimensions, and velocities of the ego vehicle and the surrounding environmental vehicles in the Frenet space. The output decisions are subsequently transmitted to the lower-level planning module, which factors in key parameters, including the total planned trajectory duration, termination speed, and lateral displacement within the Frenet coordinate system. This approach offers notable advantages, particularly in generating continuous trajectories. In comparison to trajectory planning methods detailed in references [38,39,40], our method shown in Figure 1 eliminates the need for trajectory sampling and the computational overhead of optimizing trajectories based on cost functions, consequently optimizing the trajectory planning process. Furthermore, the trajectory planning results from the lower-level planning module can be looped back to the upper-level decision-making module. They actively participate in the learning process as an integral component of the DRL reward function. This closed-loop system effectively intertwines decision making and planning, enhancing the overall stability and safety of the driving process.

The primary contributions of this paper encompass the following aspects:

(1) Integration of DRL and Frenet space: This research introduces the application of deep reinforcement learning techniques into the upper-level behavior decision-making process of intelligent vehicles. It extends the decision-making input parameters to encompass both static road mapping and dynamic obstacle information, thereby enriching the consideration dimension of decision-making process. In the lower-level trajectory planning, the incorporation of upper-layer decision-making results within the Frenet space context serves to streamline the trajectory planning procedure.
(2) Novel DRL Hybrid Reward Mechanism: A novel hybrid reward mechanism within the framework of Deep Reinforcement Learning (DRL) is proposed. This mechanism incorporates the results of lower-level planning into the upper-level decision-making process, effectively establishing a closed-loop system that iteratively refines the decision-making and planning strategies.
(3) Enhanced State Space Extraction: This paper introduces the integration of grid mapping and curve coordinate system conversion techniques into the state space extraction process for intelligent vehicle DRL algorithms. The dimensionality of this space is broadened to encapsulate size and velocity information from lane maps and environmental vehicles, which are then transformed into grid image data. This transformation streamlines the utilization of deep learning methods for feature extraction, thereby enhancing the capacity to glean relevant state information.

## 2. Methods

In this research, we delineate the distinction between behavior decision-making and trajectory planning tasks within the realm of intelligent vehicles. Behavior decision making involves utilizing scenario information to forecast the desired speed for the ego vehicle at each specific pathway point in the near future, while upholding safety. Trajectory planning entails the determination of the vehicle’s path, along with the associated lateral and longitudinal speed, within a defined time window. The results of behavior decision making are contingent upon the specific scenario and can be regarded as a Markov decision process (MDP). Addressing MDP challenges is a forte of DRL, which underpins our approach to vehicle behavior decision making. Within the DRL framework, deep neural networks are leveraged to extract both dynamic and static features from the given scenario. Reinforcement learning techniques are subsequently employed to navigate the policy space and generate optimal decision-making strategies. After a stipulated time period, based on the target path points and the prescribed vehicle speed as furnished by the behavior decision-making process, we employ polynomial programming. This technique yields smooth trajectories that meet real-time requirements and are validated as the optimal solutions, ensuring both comfort and safety. Consequently, this research advocates the utilization of the polynomial method to tackle the vehicle trajectory planning task.

The presented DRL methodology is structured into three distinct sub-processes: firstly, the determination of the state-action space; secondly, the extraction of scenario features; and lastly, the optimization of behavior strategies. This section furnishes an intricate delineation of each pivotal sub-process involved.

### 2.1. State-Action Space Determination

**Figure 2:**
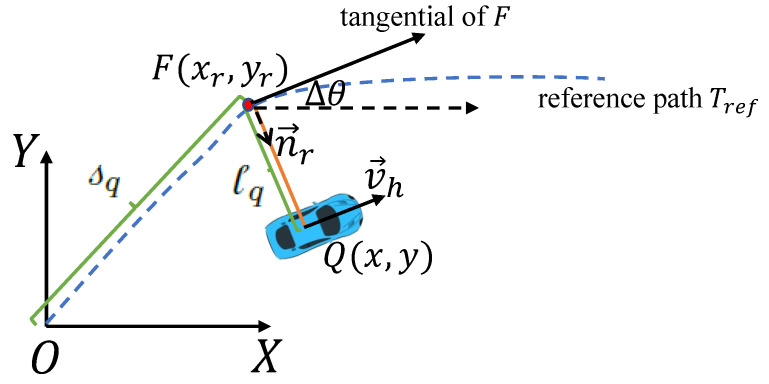
Frenet coordinate transformation.

Frenet coordinate system conversion takes the road centerline as the reference path and defines vehicle lateral offset as the vertical distance from the reference path. As shown in Figure 2, assuming that the coordinate of the ego vehicle in the Cartesian coordinate system was Q(x,y), the vehicle speed vector was ${\vec{v}}_{h}$, the reference path was expressed as $T_{ref}$, and the projection point of the vehicle position to the reference path was $F\left(x_{r},y_{r}\right)$, where the s coordinate on the reference line at *F* is then equal to the s-direction coordinate of the ego vehicle $s_{q}$. The coordinates of the ego vehicle’s location $l_{q}$ and speed in the s-direction ${\dot{s}}_{q}$ are

(1) $$\left\{\begin{array}{c} l_{q}={\vec{n}}_{r}\sqrt{{\left(x-x_{r}\right)}^{2}+{\left(y-y_{r}\right)}^{2}}\\ {\dot{s}}_{q}=\frac{\left|{\vec{v}}_{h}\right|\cos\Delta \theta }{1-\kappa l_{q}}\\ {\dot{l}}_{q}=\left|{\vec{v}}_{h}\right|\sin\Delta \theta \end{array}\right.$$

where ${\vec{n}}_{r}$ is the normal unit vector at projection point *F* on the reference path, Δθ is the yaw angle of the ego vehicle, and κ is the lane curvature at the ego vehicle’s location. The state space is composed of the ego vehicle and environmental vehicles’ features, where environmental features are represented in Frenet coordinates.

The input state of the ego vehicle is expressed as

(2) $${St}_{ego}=\left[\frac{s_{ego}-s_{0}}{s_{end}-s_{0}},\frac{l_{ego}}{\sum_{i=1}^{m}LW(m)}\right]$$

where $s_{ego}$ and $l_{ego}$ are the s and l coordinates of the ego vehicle’s position in the Frenet space, $s_{0}$ is the s-coordinate of the ego vehicle’s starting position in the Frenet space, $s_{end}$ is the s-coordinate position of the ego vehicle when it drives out of the current driving area, *m* is the number of lanes on the side of the road reference line to the direction of the ego vehicle, and LW is the lane width.

The environmental assessment encompasses vehicles located approximately in both the front and rear of the ego vehicle’s current lane, in conjunction with those in adjacent lanes situated within the ego vehicle’s sensing radius. In total, this encompasses an examination of six vehicles. Subsequently, the coordinates of these six vehicles are transformed into the Frenet coordinate system, with non-existent vehicles represented as null vectors. The composite state of the environmental vehicles is articulated as

(3) $${St}_{n}=\left[\frac{s_{n}-s_{ego}}{r_{d}},\frac{l_{n}-l_{ego}}{2LW}\right]$$

The action is selected as a combination of the duration $t_{last}$ of the ego vehicle’s trajectory, the l-direction coordinate $l_{ego}$ in the Frenet space, and the s-direction velocity ${\dot{s}}_{ego}$, i.e.,

(4) $$A=\left[t_{last},l_{ego},{\dot{s}}_{ego}\right]$$

In order to ensure the stability of the generated trajectory, the action needs to be constrained. Since the main application scenario is a vehicle driving at high speed or on the expressway, the constraint of the action’s lasting time is $t_{last}\in \left[0,6\right]$. The lateral coordinate is constrained as $l_{ego}\in \left[0,\sum_{i=1}^{m}LW(m)\right]$. The speed constraint in the forward direction is ${\dot{s}}_{ego}\in \left[10,25\right]$, which corresponds to 36–90 km/h. The above three parameters are determined as the action space for the following reason: in order to ensure the comfort of the generated trajectory, a quartic polynomial in the s-direction and a quintic polynomial in the l-direction are selected to ensure the continuity of longitudinal and lateral acceleration according to the common practice in references. In the Frenet coordinate system, trajectories can be written as

(5) $$\left\{\begin{array}{c} s\left(t\right)=a_{0}+a_{1}t+a_{2}t^{2}+a_{3}t^{3}+a_{4}t^{4}\\ l\left(t\right)=b_{0}+b_{1}t+b_{2}t^{2}+b_{3}t^{3}+b_{4}t^{4}+b_{5}t^{5} \end{array}\right.$$

In the stage of the trajectory planning task, the known quantity is the initial state of the ego vehicle $\left[s_{0},{\dot{s}}_{0},{\ddot{s}}_{0},l_{0},{\dot{l}}_{0},{\ddot{l}}_{0}\right]$. If the three parameters of the operating space can be determined, the planning state of the vehicle after the planning time $t_{last}$ can be determined as $\left[s_{ego},{\dot{s}}_{ego},0,l_{ego},0,0\right]$. The parameters of the planned trajectory can be obtained by solving Equation (5).

### 2.2. Scenario Feature Extraction Method

The extraction of scenario features relies on a deep neural network and comprises two core components: vehicle feature extraction and map scenario feature extraction.

Vehicle feature means a state sequence composed of 14 coordinate values representing seven vehicles (one ego vehicle and six environmental vehicles) at each time step. According to Equation (1)–(3), the Frenet coordinate system conversion method can be used to map the coordinates of obstacles around the ego vehicle in the road coordinate system to the Frenet space, and the calculation of vehicle scenario features can be completed.

**Figure 3:**
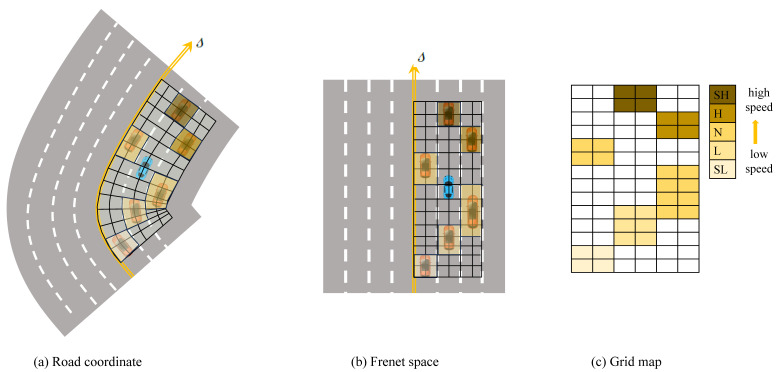
(**a**) Grid map in road coordinate. (**b**) Grid map in Frenet space. (**c**) Abstract grid map: colored grids are occupied by environment vehicles; shades of color mean speed values.

The map scenario feature involves a grid map within the Frenet coordinate system. This conversion process is illustrated in Figure 3, where a curved road in the Cartesian coordinate system (Figure 3a) is transformed into a Frenet space (Figure 3b), representing the road along the tangential (s-direction) and normal (l-direction) directions based on the road’s radius of curvature. The Frenet coordinate system conversion allows for the mapping of obstacles around the ego vehicle from the road coordinate system to the Frenet space. Since the speed and size of environmental vehicles are vital factors, they are represented via a grid map. This map is generated by converting Cartesian coordinate grids into a Frenet coordinate grid, wherein the number of occupied grids corresponds to the size of environmental vehicles and the grid colors indicate their speed values. In total, five color codes are employed, ranging from light to dark, to represent speeds less than, slightly less than, approximately equal to, slightly greater than, and significantly greater than the ego vehicle’s speed, as illustrated in Figure 3c. This approach of map scenario feature extraction offers the advantage of simplifying the state space via the introduction of fuzzy sets while considering various vehicle sizes.

**Figure 4:**
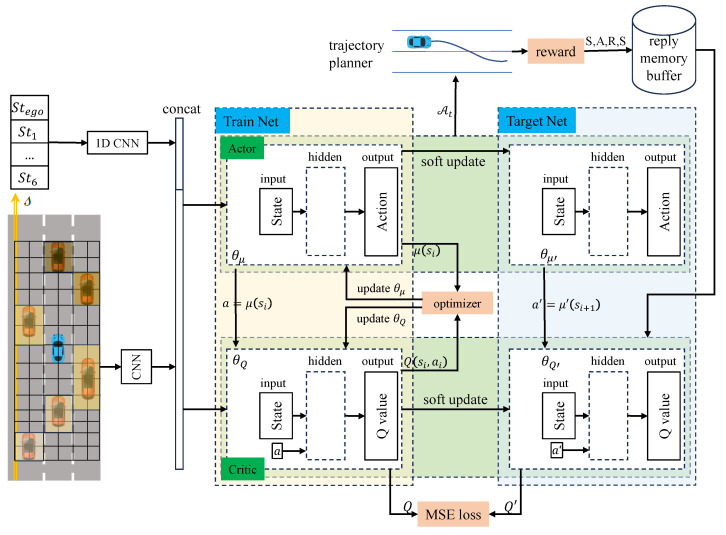
Proposed DRL method consists of deep neural network and reinforcement learning network.

The state sequence of vehicle feature extraction uses a 1D convolutional layer, while the map scenario feature employs a backbone consisting of a convolutional layer, a pooling layer, and a fully connected layer for feature extraction. Subsequently, these extracted features are concatenated and fed into the policy network, as depicted in Figure 4.

### 2.3. Action Strategy Optimization Method

The optimization of action strategies was conducted via reinforcement learning. Given that vehicle speed adaptation involves a continuous-time process, this study employs the deep deterministic strategy gradient algorithm (DDPG) as shown in Algorithm 1. DDPG stands out as an applicable choice for resolving challenges posed by continuous state-action spaces, rendering it well suited for tackling the behavior decision-making task in intelligent vehicles. Two kinds of Actor–Critic networks representing the training and the target are proposed, in which $\theta_{\mu }$ and $\theta_{\mu^{\prime }}$ are the parameters of the training Actor network $\mu \left(s|\theta_{\mu }\right)$ and the target Actor network $\mu^{\prime }\left(s|\theta_{\mu^{\prime }}\right)$, respectively, and their input is the extracted scene features. $\theta_{Q}$ and $\theta_{Q^{\prime }}$ are the parameters of the training Critic network $Q\left(s,a|\theta_{Q}\right)$ and the target Critic network $Q^{\prime }\left(s,a|\theta_{Q^{\prime }}\right)$, respectively. The Critic network is able to estimate the Q value of the state action and provide an optimized gradient for the strategy network.

(6) $$y_{i}=r_{i}+\gamma Q\left(s_{i+1},\mu \left(s_{i+1}|\theta_{\mu^{{}^{\prime }}}\right)|\theta_{Q^{{}^{\prime }}}\right)$$

where $r_{i}$ is the reward of the current action, γ is the discount factor, and $y_{i}$ is the Q value of the target Critic network.

Parameter $\theta_{Q}$ of the training network is updated using the mean-square error (MSE), and the parameter $\theta_{\mu }$ is updated using the policy gradient, i.e.,

(7) $$J\left(\theta_{Q}\right)=\frac{1}{N}\sum_{t}{\left(y_{i}-Q\left(s_{i},\mu \left(s_{i}|\theta_{\mu }\right)|\theta_{Q}\right)\right)}^{2}$$

(8) $$\nabla_{\theta_{\mu }}J\left(\mu \right)\approx \frac{1}{N}\sum_{t}\left(\nabla_{a}Q\left(s,a|\theta_{Q}\right){{|}_{s=s\left(t\right),a=\mu \left(s\left(t\right)\right)}\times \nabla_{\theta_{\mu }}\mu \left(s|\theta_{\mu }\right)|}_{s=s\left(t\right)}\right)$$

Equation (7) represents the mean-square error (MSE) loss of the training Critic network, *N* means that N transitions are randomly sampled in the replay buffer, and then the average is calculated in place of the expectation. The target networks $\theta_{\mu^{\prime }}$ and $\theta_{Q^{\prime }}$ use soft updates, i.e.,

(9) $$soft\;update:\left\{\begin{array}{c} \theta_{\mu^{{}^{\prime }}}=\tau \theta_{\mu }+\left(1-\tau \right)\theta_{\mu^{{}^{\prime }}}\\ \theta_{Q^{{}^{\prime }}}=\tau \theta_{Q}+\left(1-\tau \right)\theta_{Q^{{}^{\prime }}} \end{array}\right.$$

where τ∈(0,1) and close to 1 means the update amplitude. Rewards are allocated based on the current state and action, serving as metrics to assess the consequences of the intelligent vehicle’s behavior decision making on the subsequent trajectory planning task. The decision-making result, stemming from the reinforcement learning process, subsequently informs the planning trajectory via Equation (5). Given the potential risk of collisions or constraint violations within the vehicle’s trajectory, the reward function is categorized and detailed as follows.

(10) $$r\left(s,a\right)=\left\{\begin{array}{c} -20,income\;collision\\ -10,break\;constraint\\ 15,\;\;\;\;reach\;target\\ r_{f},\;\;\;\;feasible\;trajectory \end{array}\right.$$

Constraint violations within the generated trajectory manifest when longitudinal acceleration falls outside the range of [−5, 4], lateral acceleration exceeds [−0.8, 0.8], or the absolute curvature at any point along the trajectory surpasses 0.2 $m^{-1}$. For a feasible generated trajectory, the reward attributed to this trajectory segment can be viewed as a weighted summation encompassing aspects of comfort, deviation from the centerline at the endpoint, and driving efficiency. In essence, this is expressed as

(11) $$r_{f}=\omega_{c}r_{c}+\omega_{o}r_{o}+\omega_{r}r_{r}$$

where $r_{c}$ is the comfort reward, $r_{o}$ is the off-center line reward, $r_{r}$ is the driving efficiency reward, and $\omega_{*}$ is the corresponding weight.

(12) $$r_{c}=-\sum \left|\dddot{x}\right|\Delta t$$

(13) $$r_{o}=-\left|mod\left(l_{ego},LW\right)-\frac{LW}{2}\right|$$

(14) $$r_{r}=\sum \left|\dot{x}\right|\Delta t$$

where $\dot{x}$ and $\dddot{x}$ are the speed and jerk in the Cartesian coordinate system, Δt is the trajectory discretization time step, and mod is the remainder function. Reward function settings are diverse and dynamic, and the weight coefficients need to keep the calculated values of these three parameters within the same order of magnitude so that the final result can reflect the importance of all three factors.
**Algorithm 1:** A DDPG algorithm used to solve the behavior decision-making task of intelligent driving vehicles
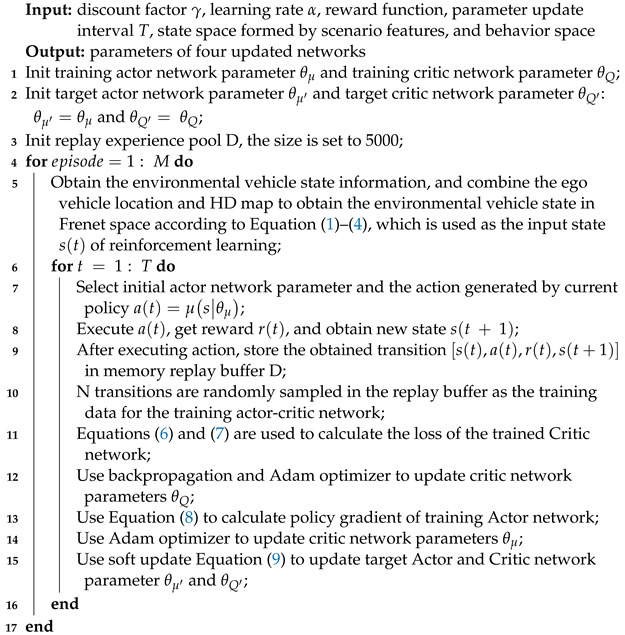


## 3. Experiments

**Figure 5:**
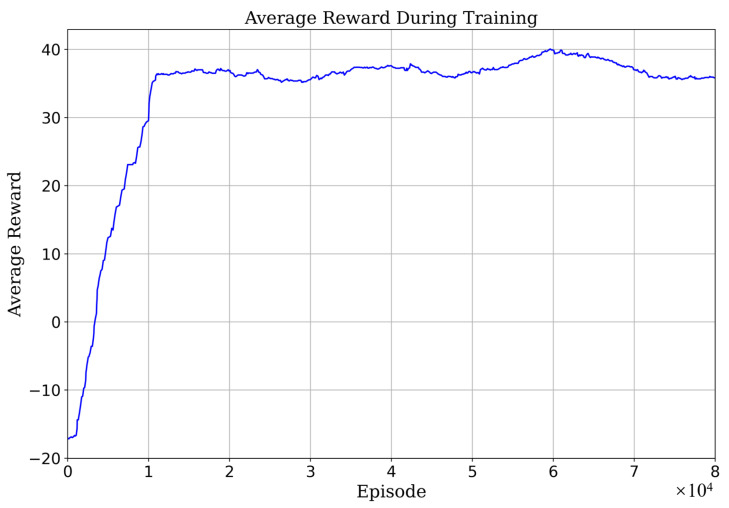
Average reward during training process.

The proposed methodology was trained and tested within a CARLA-based simulation environment featuring a four-lane highway, inclusive of various scenarios comprising both linear and curved road sections. The simulation platform assumes comprehensive knowledge of all road vehicles’ state, enabling access to vital information such as the road’s reference line positioning and lane curvatures derived from HD maps. The vehicles’ physical dimensions are determined as per CARLA-defined models, while environmental vehicle control leverages the CARLA simulator’s native rule-based autonomous driving capabilities. In each training batch, the ego vehicle commences its journey from a randomly generated starting position, progressing for a distance of 500 m or until a vehicular collision is encountered, upon which the round’s reward is computed. The pertinent hyperparameters employed during training are outlined in Table 1. At the onset of each training episode, environmental vehicles are stochastically positioned around the ego vehicle. The learning process is characterized by the average reward, as depicted in Figure 5, illuminating the method’s rapid convergence within a span of $1.2\times 10^{4}$ episodes.

**Figure 6:**
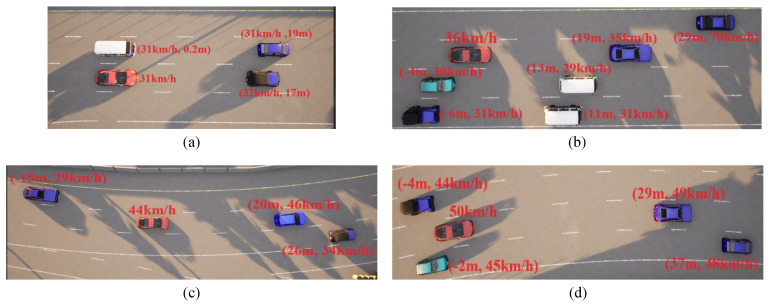
Four distinct scenarios: (**a**) straightforward linear road, (**b**) intricate linear road, (**c**) straightforward curved road, and (**d**) intricate curved road.

The training outcomes are rigorously evaluated across an array of traffic scenarios, encompassing varying traffic densities and lane configurations. Four distinct scenarios, varying in complexity and ranging from easy to challenging, have been defined: straightforward linear road, intricate linear road, uncomplicated curved road, and intricate curved road, as visually depicted in Figure 6. This illustration further annotates the initial speeds and distances of the environmental vehicles for each scenario. The ensuing test results are presented in the subsequent discussion.

**Figure 7:**
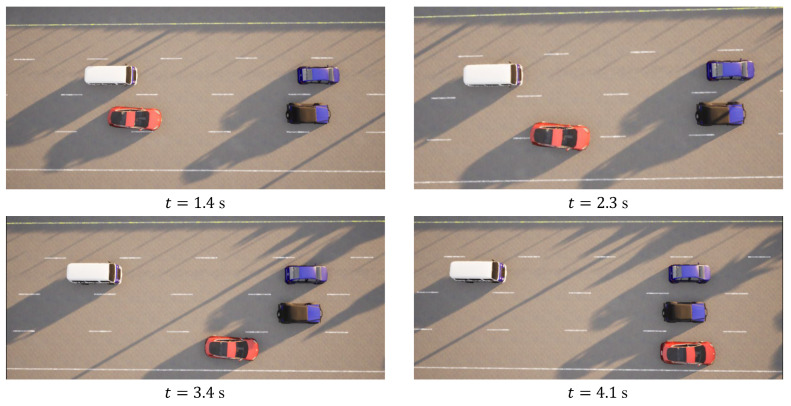
Straightforward linear road traffic flow at different moments.

**Figure 8:**
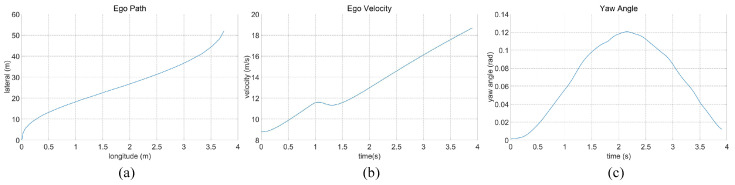
Straightforward linear road traffic analysis of ego car’s (**a**) driving trajectory, (**b**) speed, and (**c**) yaw angle.

In the case of the straightforward linear road scenario, as illustrated in Figure 6a, the roadway consists of four lanes. At the initial moment, the ego vehicle is positioned in the second lane from the right. Notably, there are three environmental vehicles directly situated ahead, left, and front left of the ego vehicle. The predetermined target speed for the ego vehicle is set at 70 km/h. The behavior decision made by the algorithm dictates a lane change to the right. The lane change process is executed over a duration of 3.9 s, commencing with an initial speed of 31 km/h at the onset of the lane change. The dynamic evolution of the traffic flow, presented in Figure 7, showcases the state transitions at distinct time intervals. The comprehensive analysis of the ego vehicle’s driving trajectory, speed profile, and yaw angle is thoughtfully elucidated in Figure 8. Upon the successful completion of the lane change maneuver, the ego vehicle proceeds to traverse to the designated end point within the newly adopted lane. Throughout this journey, both safety and efficiency considerations are diligently met. This is underscored by the average speed maintained during the entirety of the trip, which attains 48 km/h, while the speed achieved at the conclusion of the lane change approximates the preset target speed, registering at 67 km/h.

**Figure 9:**
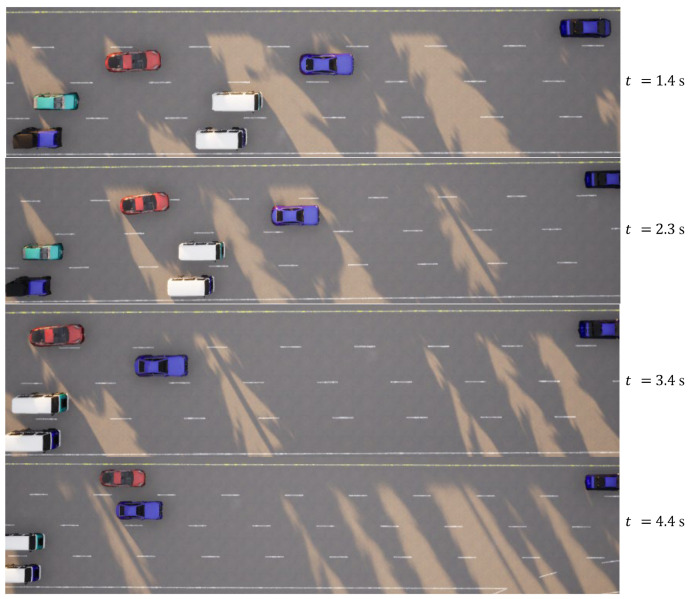
Intricate linear road traffic flow at different moments.

**Figure 10:**
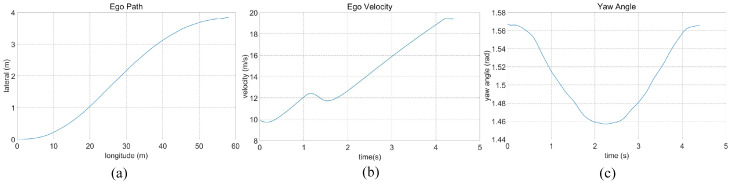
Intricate linear road traffic analysis of ego car’s (**a**) driving trajectory, (**b**) speed, and (**c**) yaw angle.

In the case of the intricate linear road scenario, presented in Figure 6b, the road configuration remains consistent with the four-lane layout. Initially, the ego vehicle is situated within the second lane from the left. The roadway scenario consists of four environmental vehicles, distributed as follows: directly in front of the ego vehicle, ahead of the ego vehicle in the right lane, behind the ego vehicle in the right lane, and ahead of the ego vehicle in the left lane. The predetermined target speed for the ego vehicle is established at 70 km/h. The algorithm prescribes a lane change maneuver, directing the ego vehicle to transition to the left lane, subsequently following the vehicle positioned ahead to the left, which is moving at a higher speed. The dynamic progression of the formed traffic flow at varying temporal junctures is meticulously delineated in Figure 9. Further elucidation is offered in Figure 10, comprising a comprehensive analysis of the ego vehicle’s driving trajectory, speed dynamics, and yaw angle. The commencement of the lane change is initiated by the vehicle at a speed of 36 km/h, with the entire lane change operation being seamlessly executed within a time interval of t = 4.4 s. Following the completion of the lane change, the ego vehicle proceeds to trail the preceding vehicle. The journey is marked by an average speed of 50 km/h, culminating in a final speed upon the conclusion of the lane change that mirrors the designated target speed, recorded at 70 km/h.

**Figure 11:**
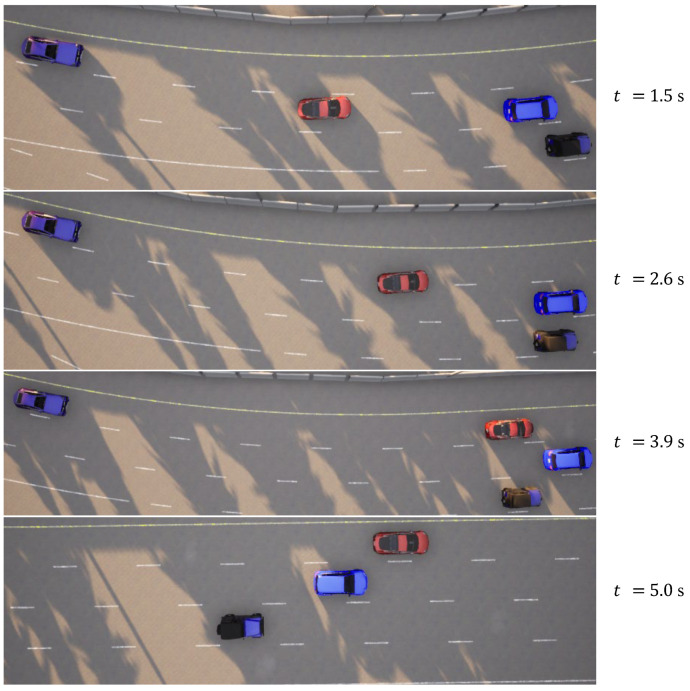
Straightforward curved road traffic flow at different moments.

**Figure 12:**
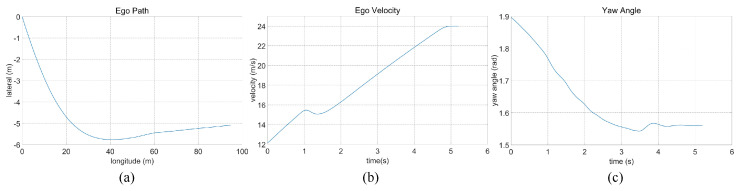
Straightforward curved road traffic analysis of ego car’s (**a**) driving trajectory, (**b**) speed, and (**c**) yaw angle.

In the case of the uncomplicated curved road scenario, characterized by three lanes and a consistent curvature, illustrated in Figure 6c, the initial conditions find the ego vehicle navigating the middle lane and encountering a sluggish-moving environmental vehicle positioned directly ahead and another in the right lane ahead. The predetermined target speed for the ego vehicle is established at 90 km/h. The algorithm dictates a left lane change maneuver. The dynamic evolution of the traffic flow, contingent upon varying temporal dynamics, is meticulously charted in Figure 11. Further elaboration is offered in Figure 12, encompassing a comprehensive analysis of the ego vehicle’s driving trajectory, speed dynamics, and yaw angle. The lane change commences within the curved road at a velocity of 44 km/h, attaining completion within a time span of 5.3 s. Subsequently, the ego vehicle maintains its trajectory within the left lane. The journey is characterized by an average speed of 65 km/h, culminating in a final speed upon the conclusion of the lane change that closely approximates the established target speed, registered at 86 km/h.

**Figure 13:**
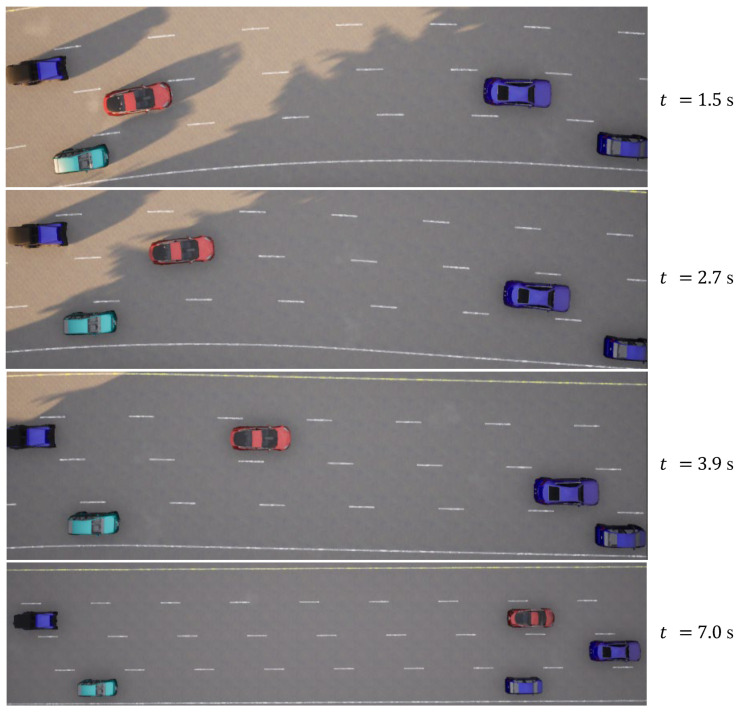
Intricate curved road traffic flow at different moments.

**Figure 14:**
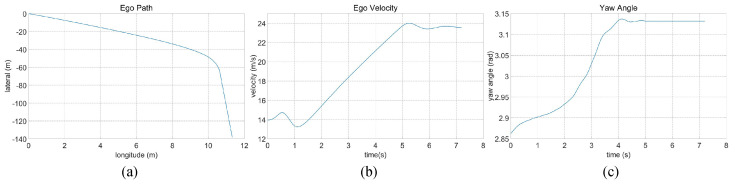
Intricate curved road traffic analysis of ego car’s (**a**) driving trajectory, (**b**) speed, and (**c**) yaw angle.

Within the intricate curved road scenario, characterized by a four-lane configuration featuring constant curvature, as depicted in Figure 6d, the initial conditions find the ego vehicle situated within the third lane from the left. The lane to the left accommodates a trailing vehicle, and the right lane features another vehicle in close proximity to the ego vehicle. Furthermore, the ego vehicle encounters slower-moving vehicles positioned ahead within the same lane and in the right lane. The pre-established target speed for the ego vehicle is set at 90 km/h. The algorithm-driven decision dictates a lane change maneuver to the left. This lane transition is executed within a duration of 4.8 s, with the vehicle commencing the lane change at a velocity of 50 km/h. The dynamic evolution of the traffic flow, contingent upon varying temporal dynamics, is meticulously charted in Figure 13. Subsequent elucidation is furnished in Figure 14, comprising a comprehensive analysis of the ego vehicle’s driving trajectory, speed dynamics, and yaw angle. Upon successful completion of the lane change, the vehicle positions itself in the second lane from the left, maintaining an average speed of 61 km/h and achieving a final speed, culminating at 86 km/h, closely approximating the established target speed.

The method proposed in this paper, which combines DDPG with the Frenet grid graph for intelligent vehicle behavior decision making and planning, is systematically benchmarked against alternative reinforcement learning techniques and planning algorithms. This comparative analysis serves to elucidate the notable advancements offered by the proposed method. To ensure a rigorous assessment, the DQN method from Reference [17] and the EM-Planner method from Reference [32] are utilized as baseline comparisons. Both the state function and reward structure used in the DQN method align with those implemented in this paper. And these models, including the EM-Planner, are subjected to the battery of four test scenarios as previously outlined. The method proposed in this paper and the two comparison methods establish the same vehicle and road models in the CARLA simulator, in which the reward function of the DQN method is set according to Equation (10), and the EM-Planner method sets the same target speed and target position of the ego vehicle, as well as it combines speed and position information of the obstacle environment vehicle transmitted to the ego vehicle in real time via a simulator, to carry out the speed planning method combining dynamic programming and quadratic programming. Specifically, for straight road scenarios, the ego vehicle’s initial speed is calibrated to 35 km/h with a target speed of 70 km/h. Conversely, for curved road scenarios, the initial speed of the ego vehicle is set at 45 km/h, with a target speed of 90 km/h. Performance metrics encompassing task completion rates, average vehicle speeds, and their respective standard deviations are scrutinized as key indicators. This comparison is executed over 100 trials for each of the four scenarios. The comprehensive results, as detailed in Table 2 where bold indicates the optimal value, distinctly demonstrate that the approach presented in this paper outperforms its counterparts across all assessed scenarios.

The comprehensive findings derived from our testing endeavors yield several noteworthy conclusions. Firstly, juxtaposed with traditional planning algorithms, the reinforcement learning methodology substantively augments the success rate of lane-change decisions and planning, particularly in scenarios involving straight roads. Secondly, in contrast to the conventional DQN approach, our proposed method markedly elevates planning performance and robustness, furnishing evident advantages. However, in the context of curved road evaluations, where the discrete nature of the DQN method is less compatible with the discontinuous trajectory planning inherent to these scenarios, the DQN approach faces a notable decline in task completion rates in comparison to the EM Planner method, which leverages kinematics and vehicle dynamics. Moreover, the robustness of trajectory planning within the DQN method remains suboptimal for these scenarios. In striking contrast, the method advanced in this paper significantly surpasses the performance of the EM Planner approach. This distinction is rooted in the capability of our approach to yield planning trajectories that not only adhere to vehicle dynamics constraints via polynomial conditions within the Frenet coordinate system, but also factor in the curvature of the road. As such, the comprehensive evaluation substantiates that the proposed approach stands as the preeminent option among the three methods under scrutiny. It should be noted that the simulations in this paper use CARLA’s own vehicle models and HD map files, which can be further obtained in Ref. [36]. Further research may require the use of customized vehicle dynamics and road models to facilitate better practical applications for environmental vehicles and ego vehicles.

## 4. Conclusions

In this research endeavor, we present a comprehensive framework for behavior decision making and trajectory planning for intelligent vehicles. This framework seamlessly combines the tenets of the DRL method and the Frenet coordinate system, effectively segmenting the driving tasks of intelligent vehicles into two core subtasks: behavior decision making and trajectory planning. The behavior decision-making component harnesses the DDPG method to equip intelligent vehicles with the ability to make informed driving decisions. This involves utilizing critical information such as relative positioning, dimensions, and velocity of the ego vehicle in relation to environmental vehicles within the Frenet coordinate space as inputs to the DDPG model. The outcome of this decision-making process then serves as essential input for the trajectory planning subtask, augmenting it by providing a comprehensive picture of parameters like the planned trajectory’s lasting time, final velocity, and lateral displacement. The score of the obtained trajectory can also be used as part of a reward to participate in the optimization of the DRL method. Notably, extensive experimentation within the CARLA simulator substantiates the merit of our proposed approach. It showcases robust feasibility, stability, and efficiency across diverse driving scenarios, surpassing the baseline DRL methodology and traditional vehicle trajectory planning algorithms.

Looking ahead, the avenue for future research could encompass broadening the scope of applications for our proposed method, thereby enabling its utilization in a wider array of scenarios. Furthermore, the pursuit of more precise mathematical and physical models for vehicle control is encouraged, as these would furnish enhanced guarantees regarding the real-world viability of our proposed method. Deep learning methods for extracting environmental features and reinforcement learning methods for decision making can use better performance schemes that have been proven in the literature.

## Figures and Tables

**Table 1:** Hyperparameter in training process.

Hyperparameter	Symbol	Value	Comment
Discount factor	γ	0.99	Algorithm 1 input
Batch size		128	Algorithm 1 input
Replay buffer size	*D*	5000	Algorithm 1 input
Parameter update interval	*T*	5	Algorithm 1 input
Comfort reward weight	$r_{c}$	5	Equation (11)
Off-center line reward weight	$r_{o}$	1	Equation (11)
Driving efficiency reward weight	$r_{r}$	0.2	Equation (11)

**Table 2:** Performance metrics for different methods at four mentioned scenarios.

Scenario	Algorithm	Task Completion Rate (%)	Mean Velocity (km/h)	Standard Deviation
straightforward linear road	DQN	96.8	45.6	0.92
	EMP	94.6	46.4	0.77
	ours	**99.3**	**47.5**	**0.52**
intricate linear road	DQN	95.9	46.3	0.82
	EMP	93.2	48.7	0.77
	ours	**98.6**	**50.0**	**0.58**
uncomplicated curved road	DQN	74.3	56.3	2.81
	EMP	90.6	57.8	1.05
	ours	**97.8**	**64.2**	**0.66**
intricate curved road	DQN	72.7	48.4	2.96
	EMP	87.3	56.6	1.12
	ours	**96.5**	**60.3**	**0.69**

## Data Availability

Data are contained within the article.
